# Supplementary material for: Implementing the CRISPR/Cas9 Technology in Eucalyptus Hairy Roots Using Wood-Related Genes
Source: Int J Mol Sci. 2020 May 12;21(10):3408. doi: 10.3390/ijms21103408 (PMC7279396; doi:10.3390/ijms21103408)
Supplement: Supplementary file 1 [file ijms-21-03408-s001.zip › 14 avril Supplementary Figures CRISPR CCR_final.pptx]

## Slide 1
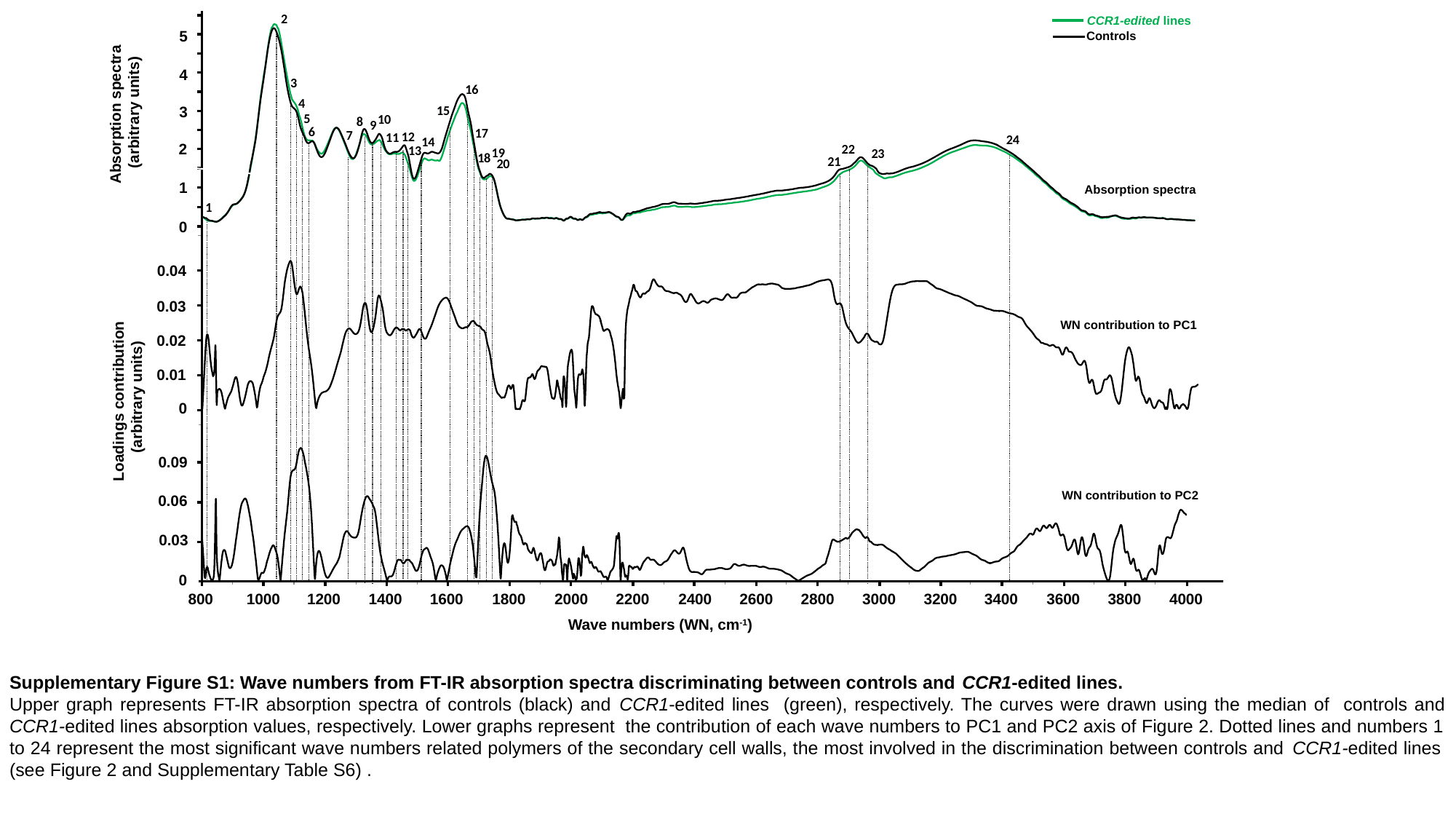

CCR1-edited lines
5
Controls
4
Absorption spectra
 (arbitrary units)
3
2
1
Absorption spectra
0
0.04
0.03
WN contribution to PC1
0.02
0.01
Loadings contribution
 (arbitrary units)
0
0.09
WN contribution to PC2
0.06
0.03
0
800
1000
1200
1400
1600
1800
2000
2200
2400
2600
2800
3000
3200
3400
3600
3800
4000
Wave numbers (WN, cm-1)
Supplementary Figure S1: Wave numbers from FT-IR absorption spectra discriminating between controls and CCR1-edited lines.
Upper graph represents FT-IR absorption spectra of controls (black) and CCR1-edited lines (green), respectively. The curves were drawn using the median of controls and CCR1-edited lines absorption values, respectively. Lower graphs represent the contribution of each wave numbers to PC1 and PC2 axis of Figure 2. Dotted lines and numbers 1 to 24 represent the most significant wave numbers related polymers of the secondary cell walls, the most involved in the discrimination between controls and CCR1-edited lines (see Figure 2 and Supplementary Table S6) .

## Slide 2
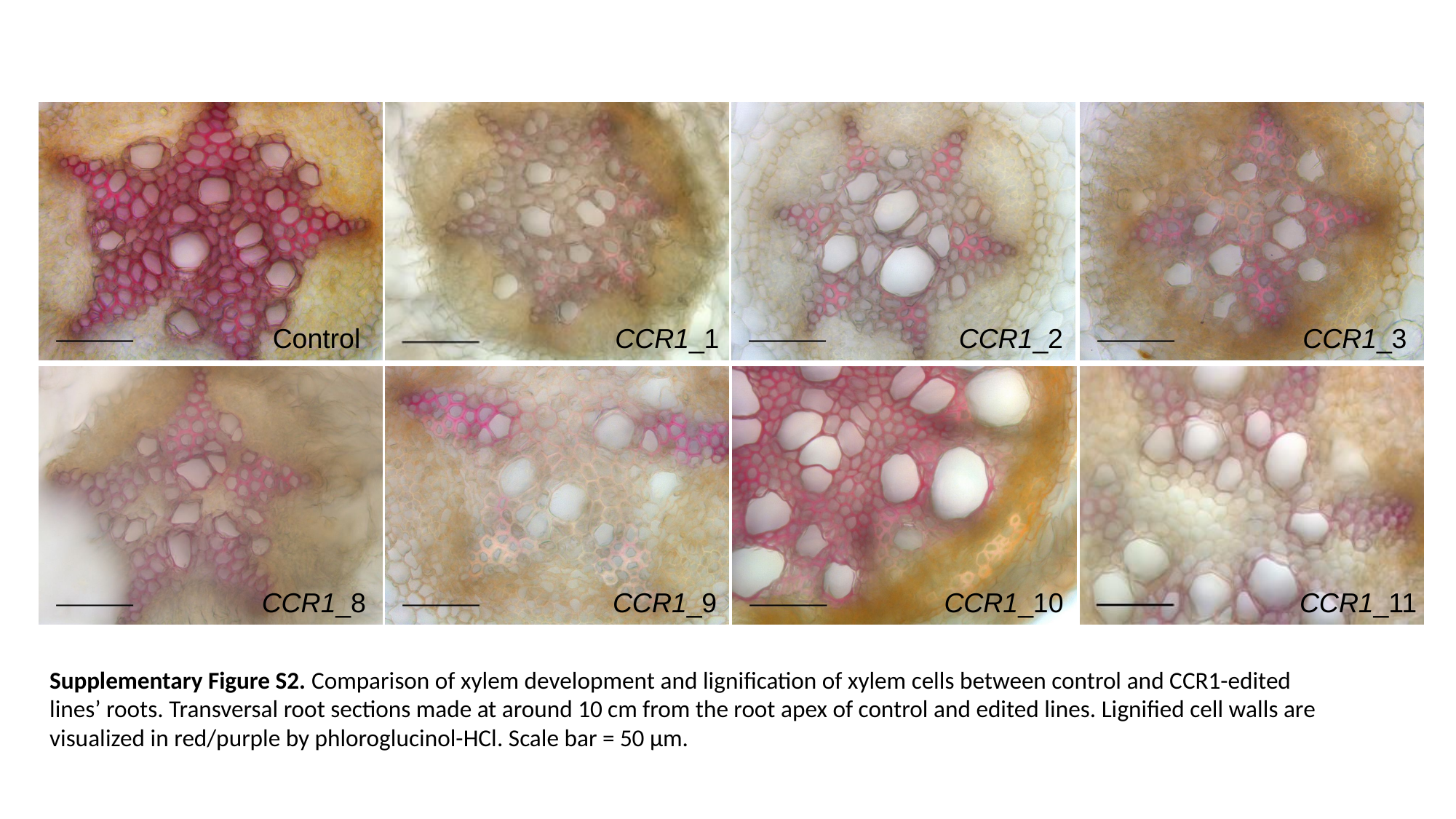

Control
CCR1_1
CCR1_2
CCR1_3
CCR1_8
CCR1_9
CCR1_10
CCR1_11
Supplementary Figure S2. Comparison of xylem development and lignification of xylem cells between control and CCR1-edited lines’ roots. Transversal root sections made at around 10 cm from the root apex of control and edited lines. Lignified cell walls are visualized in red/purple by phloroglucinol-HCl. Scale bar = 50 µm.
